# Impact of an Individualized Cognitive Training Intervention in Preschoolers from Poor Homes

**DOI:** 10.3390/ijerph17082912

**Published:** 2020-04-23

**Authors:** Federico Giovannetti, Marcos Luis Pietto, María Soledad Segretín, Sebastián Javier Lipina

**Affiliations:** 1 Unidad de Neurobiología Aplicada (UNA), CEMIC-CONICET, Buenos Aires C1431FWO, Argentina; marcos.pietto@gmail.com (M.L.P.); soledadsegretin@gmail.com (M.S.S.); 2 Laboratorio de Inteligencia Artificial Aplicada, Instituto de Ciencias de la Computación, FCEyN-UBA- CONICET, Buenos Aires C1428EGA, Argentina

**Keywords:** executive functions, cognitive control, poverty, cognitive enhancement, intervention, training

## Abstract

Over the last few decades, different interventions were shown to be effective in changing cognitive performance in preschoolers from poor homes undertaking tasks with executive demands. However, this evidence also showed that not all children included in the intervention groups equally increased their performance levels, which could be related to individual and contextual variability. The present study aimed to explore the impact of a computerized cognitive training intervention with lab-based tasks in preschoolers from Unsatisfied Basic Needs (UBN) homes under the consideration of their baseline performance. In the context of a randomized controlled trial design, different interventions were administered to children according to their baseline performance in a variety of cognitive tasks (i.e., executive attention, inhibitory control, working memory, and planning demands). The results showed different patterns of impact on performance depending on the experimental group, supporting the importance of considering individual and contextual differences in the design of interventions aimed at optimizing executive functions in poverty-impacted sample populations in early stages of development.

## 1. Introduction

### 1.1. Executive Functions and Cognitive Interventions in Poverty

Executive processes, such as attention, inhibitory control, working memory, cognitive flexibility, and planning, can be defined as a complex set of abilities involved in the regulation of emotions, thoughts, and behaviors during an objective-oriented action, such as those involved in learning contexts [1,2]. The influence of early experiences on emotional, cognitive, and social processing is associated with a child’s opportunities for social and educational inclusion during the first two decades of life [3,4,5,6] 

The sensitivity of each child to the material and symbolic characteristics and resources in their developmental contexts is highly variable [7]. Consequently, attention, inhibitory control, working memory, and flexibility processes vary between individuals from a very early stage of development [8,9,10,11]. In particular, the perspective of the Relational Developmental Systems (RDS) conceptualizes these individual differences in a complex way, in which development implies bidirectional and interdependent associations between events at different levels of organization (i.e., genetic, epigenetic, cellular, neural, cognitive, behavioral, and contextual) [12]. 

Experimental and applied research in this area showed that adverse experiences linked to poverty in the early stages of development were associated with changes in different aspects of cognitive processes [10,13,14]. Studies with infants, preschoolers, and school-aged children indicated associations between family socioeconomic status (SES) and performance in tasks requiring attention, inhibitory control, working memory, flexibility, and planning [15,16,17,18]. In addition, this evidence indicated that SES was usually associated with lower performance in language and mathematics [13,19,20], and that executive functions mediate such associations [21].

Over the last few decades, different types of interventions were shown to be effective in changing cognitive performance in tasks involving executive demands in preschoolers from poor homes. Some interventions were derived from multimodular intervention programs [22,23,24,25], and other experimental approaches evaluated the impact of cognitive training activities embedded into school curricula [26,27,28] and parenting interventions [29,30], with only a few involving individual manual or computerized activities in which inhibitory control and attentional processes were trained [31,32]. However, the evidence also showed that not all children included in the intervention groups increased their performance levels equally, possibly due to individual and contextual factor variability [32,33]. 

### 1.2. Individual Differences in the Impact of Cognitive Interventions

The RDS approach proposes the need for person-oriented research designs to understand the influence of individual differences on development. This perspective defies the idea of homogeneity within samples [34]. Thus, considering all subjects in a sample to be homogeneous could mask intra- and inter-individual differences in various factors, such as cognitive performance, trajectories, and contexts. Exploring individual differences within populations could support the design of adapted interventions for children with particular needs or those at risk in any dimension by identifying the individual characteristics of each participant at a certain developmental period [35,36,37].

In recent years, cognitive performance was used to group children into performance groups or profiles. For example, Dauvier, Chevalier, and Blaye [38] assessed the existence of different performance profiles in a sample of children who were administered multiple cognitive flexibility and working memory tasks. Each profile was determined based on the response patterns of each child in the flexibility task. Five different performance profiles were generated (“random switch”, “perseveration”, “mainstream”, “efficient set-shifting”, and “strategy change”) and each one was related to different ways of solving the task and performance levels in the working memory (WM) task. Lucenet and Blaye [39] divided a sample of five-year-old children based on the median individual performance and found qualitative differences (reactive vs. proactive control) for each group in the way that they solved an attentional task. Also, in a study conducted by Fracchia and colleagues [40] in children from diverse SES backgrounds, different performance groups conducted planning and working memory tasks, where the groups were estimated based on the child’s individual performance in each trial with regard to the performance of the whole sample for the same trial and performance in previous trials. In addition, differences were found in the composition of those profiles according to SES background.

Few training studies considered the role of individual differences in baseline cognitive performance on the impact of training, with most of them carried out using adult populations [41,42,43,44]. A recent publication assessed an adult sample [45] and analyzed data from three studies to identify factors predicting cognitive training impact. Baseline cognitive performance was the only factor that predicted cognitive training gains. Some studies with children in middle childhood showed the same association [41,46,47,48]. In both cases, children with lower baseline cognitive performance were those who benefited the most from the interventions. This effect was defined by the authors as a *compensation account,* whereas the opposite effect (by which those who benefited the most were the highest performing individuals) was named as the *magnification account.* Nonetheless, both tendencies were mostly studied in adult samples with regard to the domain of working memory interventions and middle- or high-SES samples. 

The present study aimed to explore the impact of a computerized cognitive training intervention with lab-based tasks in preschoolers from Unsatisfied Basic Needs (UBN) homes, under the consideration of their baseline performance. Different interventions were administered to children according to their baseline performance in a variety of cognitive tasks. A second aim was to explore differences in the impact of the intervention for each training scheme. We expected to see a training effect in the intervention groups in comparison to the control groups. Further, we expected to see differences in the intervention effect depending on the different interventions implemented for each performance group. However, we could not predict the direction of these predicted effects because of a lack of evidence in the literature regarding this. The results presented in this article correspond to a larger study, of which some results were previously published [49].

## 2. Materials and Methods 

### 2.1. Study Design

**Figure 1:**
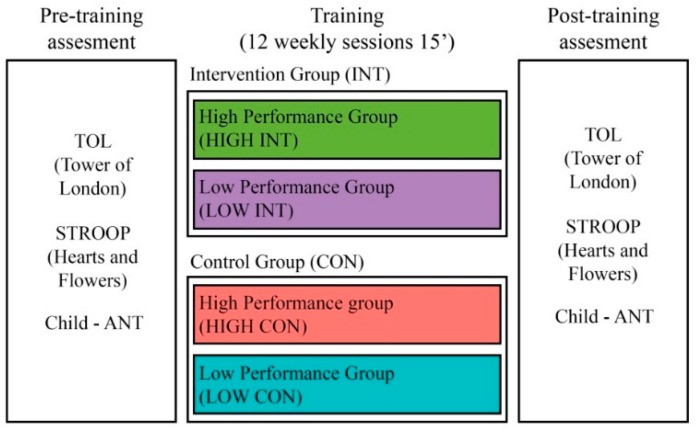
Study design. Note. TOL = Tower of London task, STROOP = Stroop Task, Child-ANT = Attentional Networks Tasks, INT = Intervention group, CON = Control group.

We implemented a quasi-experimental longitudinal controlled and randomized design, classified as an efficacy study [50] (Figure 1). Children were distributed into intervention (INT) and control (CON) groups and classified as high- and low-performing groups based on the children’s baseline performance in three tasks, i.e., inhibitory control, working memory, and planning. Differential training menus were designed for each group. Training consisted of a total of 12 sessions in which children performed 3 different activities (4 sessions for each one). After training implementation, the same cognitive tasks used in the pre-training stage was administered to each group. 

### 2.2. Participants

All participants attended a public kindergarten in Buenos Aires City. The school schedule was full-time (from 8:45 am to 4:00 pm), included three meals (breakfast, lunch, and afternoon snack) and nap time. Parents or legal caregivers attended an informative talk about the characteristics of the study and gave written consent to participate. 

The original sample consisted of 119 children. A total of 37 children were excluded from the analyses due to developmental disorders (n = 3), not starting the intervention (n = 9), not finishing the 12 intervention sessions (n = 9), not completing the post-training assessments (n = 13), or error made by the researchers in the group assignment (n = 2). Lack of completion of intervention sessions and post-training assessments was due to high rates of absenteeism. The final sample for this study was composed of 82 children (38 girls; median age: 5.25, Q25: 5.1, Q75: 5.6).

All procedures described in this manuscript followed national and international research procedures and norms and were reviewed and approved by the Institutional Review Board (CEMIC, Protocols N° 682, and 961).

### 2.3. Sociodemographic Information

A questionnaire was administered to one parent of each child in a school room to obtain information on SES and family living conditions [18,31]. A total socioeconomic score (NES) was determined based on the following criteria: (1) higher parental educational level (values between 0 and 12: no education = 0; incomplete primary school = 1; primary school degree = 3; incomplete high school = 6; high school degree = 9; incomplete technical studies = 9; complete technical degree = 10; incomplete college studies = 10; college degree or higher = 12); (2) higher parental occupation level (values between 0 and 12: unoccupied = 0; unstable worker = 1; unskilled laborer = 2; skilled laborer = 4; small autonomous producer = 6; administrative employee = 7; technical professional = 8; small business owner = 10; professional = 11; company manager = 12); (3) dwelling characteristics (values between 3 and 12 based on type of house, floor, ceiling, and external wall materials, access to drinking water, bathroom with sanitation system, and home property); and (4) overcrowding (values between 0 and 9 based on the amount of people and rooms: 1 to 2 people per room = 9; 2.01 to 4 people per room = 6; 4.01 to 6 people per room = 3; and more than 6.01 per room = 0). A home was considered to have UBN if at least one of the following indicators was identified: (1) inappropriate dwelling (housing); (2) absence of waste discharge system in household; (3) overcrowding (more than 3 people per room); (4) presence of school-aged children not attending any educational system; and (5) head of household with incomplete secondary schooling with more than four dependents.

Additionally, the questionnaire included items to describe children´s general health condition and history of developmental disorders, sleep quality, and nutrition. Sleep quality scores were 1–5 (5 = highest) based on sleep onset and maintenance, somnolence, and breathing problems [51,52]. A nutrition score was computed using information about type of nutrients (i.e., energy, proteins, vegetables, fruits, and dairy) and daily intake (i.e., breakfast, lunch, snack, dinner, and three snacks per day). 

Finally, we administered the Child Behavior Questionnaire (CBQ) [53] to measure three temperamental factors, namely, *Surgency,* characterized by high impulsivity and activity and low shyness, *Negative Affect,* relating to high levels of sadness, fear, anger, frustration, and restlessness, and *Effortful Control,* relating to high levels of inhibitory and attentional control. This questionnaire was administered in an individual interview to the caregiver.

### 2.4. Evaluation Procedures

#### 2.4.1. General Aspects

All activities were implemented in a table of 1.30 × 0.5 m in a kindergarten room. In both the evaluation and training sessions, a maximum of 3 children were seated at the table. Each child was assessed or assisted by a research assistant who was blind to the design and hypotheses of the study and was previously trained for task administrations. Children were separated by a folding wooden screen to avoid distractions and interferences during the administration of tasks and activities. All tasks and activities were performed on a tablet (Samsung Galaxy tab E with a 9.6” screen) placed at a distance of about 30 cm away from the child.

Running studies in the school context are recommended as good practice [54] and were proven to be feasible and reliable in the field [28,31,32,55].This type of approach allowed us to come and go between the mechanistic and efficacy study questions while testing the lab-based measures in the children’s daily real-world contexts [50]. In addition, the friendly context allowed us to access a bigger sample and increase the amount of time spent assessing and training the participants.

#### 2.4.2. Cognitive Measures

Only cognitive measures implied in this manuscript are described in this section. Baseline performance in Tower of London was utilized to distribute children homogeneously between experimental groups (control and intervention). In this work, analysis of the impact of the intervention was conducted only for Child Attentional Networks Test (ANT) and Stroop task. 

*Child ANT:* A version of the Attentional Networks Test for children (ANT) [56] was used to assess different aspects of attentional processing and inhibitory control (Cronbach’s alpha = 0.9). The adapted version (also used in [31,55]) consisted of a screen with two buttons on the sides and a fixation cross in the center. After the appearance of the cross, different cue conditions could occur for 150 ms: No Cue; Central Cue, which appeared as a white oval figure in the middle of the screen; Double Cue, which involved one white oval in the upper screen and another in the lower middle screen; or Spatial Cue, which would appear in the same place as the subsequent stimulus. The cues were followed by a 450 ms between the post-cue time and the stimulus. The stimulus consisted of a row of 5 figures (fish, mouse, or bird) and the child had to press the button on the side according to which the figure in the middle was pointing to. Sometimes, the figure in the middle would be pointing to the same side as the rest (congruent) and sometimes to the opposite side (incongruent). This task was divided into three blocks of 32 trials in which the stimulus appeared for 1700 ms with a positive or negative feedback of 2000 ms and an inter-trial time of 1000 ms. This task included a practice block of 8 trials that could be passed if the child had 5 correct trials; otherwise, children repeated the practice block once more. Dependent variables for this task included *omitted trials* (i.e., the number of trials without response), *proportion of correct trials* (correct vs. total trials), *performance* (proportion of correct responded trials, i.e., correct vs. answered trials), *reaction time* (RT), *alerting network* (RT for no-cue trials minus RT for double-cue trials), *orienting network* (RT for central-cue trials minus RT for spatial-cue trials), and *executive network* (RT for incongruent trials minus RT for congruent trials).

*Hearts and Flowers (Child Stroop task):* The Hearts and Flowers task is a Stroop-like task for children [2,57] that taps inhibitory control and cognitive flexibility processes (Cronbach’s alpha = 0.82). In the version used for this study [31], the screen presented a similar distribution to that of ANT with buttons on the sides and a fixation cross in the middle. The task had three blocks of trials. In the congruent block, a strawberry would appear on either the left or right side and the child was told to press the button located on the same side of the stimulus. In the incongruent block, a slice of watermelon would appear on the left or the right side and the child was told to press the button located on the opposite side of the stimulus. Finally, in the mixed condition, both stimuli would appear and the child would have to remember both rules. The stimulus would appear for 1500 ms, with 1500 ms of inter-trial time. The first and second blocks consisted of 12 trials plus 6 practice trials, while the third block consisted of 24 trials with no practice. Dependent variables for this task included *omitted trials* (i.e., the number of trials without response), *proportion of correct trials* (correct vs. total trials), *performance* (proportion of correct responded trials, i.e., correct vs. answered trials), *reaction time* (RT) for each block, and *spatial incompatibility effect RT* (RT of incongruent trials in the mixed block minus RT of congruent trials in the mixed block).

*Tower of London Task (TOL).* This task was designed to study planning processes [58]. For this study, we used a similar version to the one used by Owen and colleagues [59] with a problem based on suggestions by Kaller and colleagues [60]. The task presented three holes in the sand with descending sizes. The first hole can fit three balls, the second two balls, and the third only one ball. Three balls were sorted into the holes, forming the initial model. In the superior left corner, a similar model was presented with a different ball configuration as the end goal, which could be achieved in a determined number of minimal moves following certain rules: (1) Children could move only one ball at a time and (2) they could only move the top ball in each hole. The balls were moved in a “drag and drop” way. The number of required moves was stated before each trial and increased progressively from 1 to 7. The task was interrupted after 5 consecutive incorrect trials. Dependent variables considered for this task included *performance* (the proportion of correct trials, i.e., correct vs. total trials) and *planning time* (time from the start of the trial until the first ball was placed in any hole).

### 2.5. High- and Low-Performing Group Classification

Children were classified into two groups, i.e., high-performing group (HPG) and low-performing group (LPG) according to their baseline performances in the Stroop, Tower of London (TOL), and Corsi blocks tasks. This classification aimed to identify low and high performers in each task in order to apply different intervention schemes based on the complexity of the task and the activities each group was required to solve. LPG was defined by children performing below the median of the correct trials, with children above the median assigned to the HPG. 

### 2.6. Intervention

Each intervention menu consisted of 12 training sessions of 15 minutes each, with an average time between sessions of 7.61 days (SD = 0.99). As in the pre- and post-intervention assessments, each child was assisted by a research assistant (trainer) to ensure the child’s engagement with the task and to assist them when difficulty was experienced.

#### 2.6.1. Intervention Group Activities

Difficulties in the Intervention group (INT) were identified following a problem-solving framework [61], which was also used by the research group in previous studies [32,62]. Each trial resolution was conceptualized as involving four steps, namely, problem representation, planning, execution, and evaluation. Different approaches were taken into consideration depending on the difficulties observed by the trainer. For example, if children showed difficulty accomplishing the task, they were encouraged to continue practicing, when the task was misunderstood, trainers repeated the instructions as many times as necessary, and when an impulsivity-related error was detected, children were taught to wait and to think carefully before acting. Finally, activities followed two main principles, i.e., the inclusion of new challenges with increasing difficulty and repeated practice [63]. Participants in the training group were administered three activities designed for cognitive training. Each training activity was administered for two consecutive sessions in the order of *inhibitory control activity, working memory activity,* and *planning activity.*

The *inhibitory control activity* consisted of a Stroop-like task where children had to tap a button (right or left) when a pre-potent stimulus was given. Participants were presented a screen where stimuli would appear one at a time (a plane or a rocket of different colors) on one side of the screen, pointing either to the right or to the left. The child was required to establish the direction of the stimulus by controlling different conditions. In the congruent condition, a yellow plane or rocket appeared and the child was told to press the button to which the plane was pointing. In the incongruent condition, a red plane or rocket appeared and the child was told to press the opposite button to which the stimulus was pointing. Finally, in the mixed condition, both yellow and red stimuli appeared. In advanced levels, some distractors appeared (other flying objects, such as paper planes, balloons, or paper planes of other colors). Before congruent and incongruent conditions, the rules were explained with a short video. The difficulty of the activity was determined according to the condition, the time available to answer, and the presence of distractors.

The *working memory activity* [55] was designed to measure working memory for visual patterns and was based on the Self Ordered Pointing Task (SOPT) [64,65]. Various items (i.e., cards with different pictures and colors) were presented within a 4 × 3 rectangular grid and children had to choose one item. After this, the items disappeared and reappeared in a different order. Children had to choose a different item than the one selected in the previous trial. In each trial, a constant number of items appeared. The trial ended when all the items had been selected or when the child selected an incorrect item (one that had previously been selected). The number and difficulty of the items increased as the children won more trials. The activity started with trials with a low number of items (three) to recall; if the child answered correctly three consecutive time, the number of items was increased to 4. If they answered wrong in 4 consecutive trials, the level decreased by one. According to a study performed by Cragg and Nation [66], adults and children committed more errors in the SOPT task when it contained abstract items than when the objects carried meaning. In the version used in this study, the activity started with items composed of simple images (e.g., a plain color background and a cartoon character) and a low number of items to recall and advanced to more complex and numerous items (e.g., items with images containing a plain color background and a cartoon character in an abstract shape). Finally, this activity allowed the use of mnemonic rules or strategies to remember all items, which was usually performed by the subjects sorting the list of objects into categories (e.g., if there were two items with a red background and another two with a blue background, one could first select the two blue items and then two the red ones) [31]. Participants were encouraged to use this strategy when they started to experience difficulty.

The *planning activity* was an adaptation of the Dog–Cat–Mouse task [67]. The screen showed a square with a diagonal and three “houses” sorted in the four corners. Three characters were sorted into one house each in different corners and the aim was to guide them to their houses in a determined number of moves. Children were given three rules: (1) the characters could be moved one at a time and could only be moved to an empty corner, (2) they could only be moved through the “paths” (sides and diagonal), and (3) characters could not share house. As the activity progressed, the number of movements required to reach the objective increased, thereby increasing the path length. For this study, we controlled a variety of problem parameters in addition to the path length, including the use of the diagonal (which would make a problem easier), the number of possible paths, whether the first movement allowed two or three moves (the probability of making a bad choice diminished with two moves), and search depth (the number of moves necessary to guide the first character to its house). This training activity consisted of two phases, namely, a free exploration phase, in which trials were considered correct if the child guided all characters to their houses with an unlimited number of moves, which ended when the child completed all of their trials, and a restricted movement phase, in which the child was given a number of movements in which the trial had to be solved. After three consecutive errors, the activity stopped and started again from where the child started at the beginning of the session. 

#### 2.6.2. Control Group Activities

Participants assigned to the control group (CON) played 3 different games available for free download from Google Play Store which were not created for cognitive training purposes. *Bubble shooter* required the child to destroy a bunch of bubbles placed at the top of the screen by directing and shooting a bubble of the same color from the bottom. In the *Painting* game, the child was asked to paint a wide variety of animals, cars, and objects with the fingers. Finally, the *Dots* game consisted of an array of colored circles that disappeared when the player connected circles with a similar color. These activities were not intended to generate training effects in participants due to the fact that they were not created for cognitive training purposes and no evidence suggests that they tap or train the cognitive processes we proposed for the intervention group.

### 2.7. Targeted Training Menus

**Figure 2:**
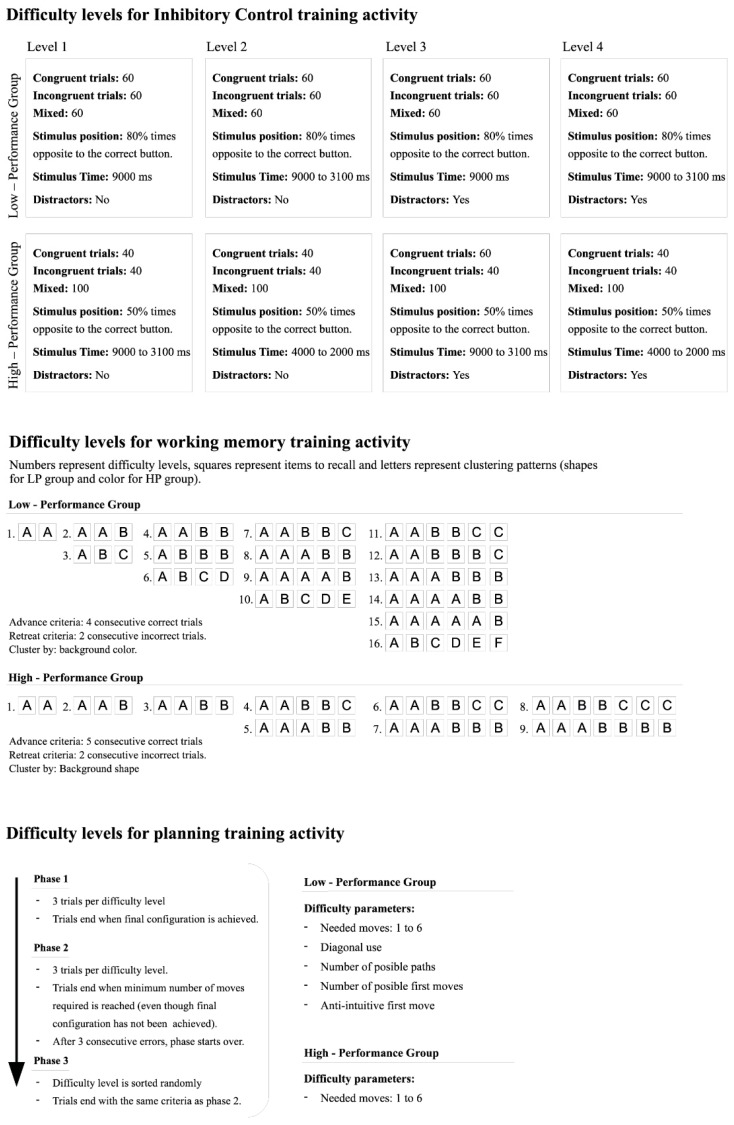
Training schemes and difficulty levels for each training activity and performance group.

Based on the performance groups created after the pre-training assessment, different targeted training menus were designed for each group. Thus, for each training activity two menus with different levels of difficulty were designed (see Figure 2).

#### 2.7.1. Inhibitory Control Menus

For the inhibitory control activity, two training menus were designed, each with 4 levels. Children played until they made 8 nonconsecutive errors and then started again from the beginning of the stage. The advance criteria for each group varied. In order to get to level 2, the LPG had to complete the congruent and incongruent conditions (60 trials each), whereas the HPG had to accomplish both conditions (40 trials each) and 20 mixed trials. To get to the following levels, the LPG had to complete the congruent and incongruent conditions plus 40 mixed trials (120 trials in sum); and the HPG had to accomplish both conditions and 50 mixed trials (130 trials in sum). In addition, the number of trials per condition, trial time, position, orientation, and the addition of distractors were considered when designing the targeted menus. In LPG, the congruent and incongruent conditions were larger than in the HPG. Further, in the congruent condition, 80% of stimulus (plane or rocket) appearances were on the opposite side to which it was pointing, whereas in the incongruent condition, the stimulus appeared 80% of the time on the same side to which it was pointing. For the HPG the position of the stimulus was set randomly. This was done in order to extend the time to which LPG was exposed to inhibitory control trials before facing the mixed condition, giving a smoother difficulty slope for LPG than for HPG. The time of stimulus presentation for each trial in the LPG group was maintained at 9000 ms in the first level and then decreased gradually to 3000 ms. For the HPG, the trial time on levels 1 and 3 started at 9000 ms and decreased gradually to 3000 ms, while in levels 2 and 4, it started at 4000 ms and decreased to 2000 ms. In both groups, distractors were added for levels 3 and 4.

#### 2.7.2. Working Memory Menus

For the working memory activity, two training menus were designed. A set of trials with growing difficulty was defined for each performance group considering the number of items to recall, their complexity, and the number of possible chunks. In both performance groups, children were assisted in the use of the chunking strategy by the trainer. For the LPG, the items had two features, i.e., the background color (6 different themes), and a character (5 different themes). The background color was considered the grouping feature for the clustering strategy. Increasing difficulty was organized in levels. The first one consisted of two items with the same color where, if the child did well in 4 consecutive trials, he or she would advance to the next level with 3 items, one of them of color A and the other two of color B, providing the chance to advance in the number of items to recall while still using the “assistance” of the clustering strategy. The next level had 3 items to recall with three different background colors. This sequence was repeated for the next levels with a growing number of items and number of chunks. If two consecutive errors were made, the activity regressed by one level. Overall, the scheme involved recalling 2 to 6 items. For the HPG, the items had three features, including color (6), shape (6), and a character (five different themes). The shapes chosen were complex (e.g., octagons and pentagons), which were intended to be much more difficult to name and give meaning to than the background colors. Difficulty levels for the HPG were steeper than in the LPG. For example, in the LPG, the 4 item levels went from an A-A-B-B color sorting to an A-A-A-B configuration and then to A-B-C-D; in the HPG there was only one 4 item level, meaning that the child had to go from 4 items to 5 without any intermediate level. In addition, HPG individuals were required to complete five trials to level up. This scheme involved recalling 2 to 7 items.

#### 2.7.3. Planning Menus

For the planning activity, two training menus were designed, each with different difficulty levels composed of some of the problem parameters mentioned in the previous section. For LPG, difficulty levels were based on the path length, the number of first possible movements, the use of the diagonal, the search depth, and whether the first movement was anti-intuitive or not. All difficulty levels were ordered around the number of minimum movements. For example, within three movements trials, five levels were created by combining each problem parameter and ordered according to the literature from easiest to hardest [67]. This diagram was used for both phases from one to six movements. For the HPG, difficulty levels were based only on the path length, leaving only six levels for each phase.

### 2.8. Statistical Procedures

The normality of sample measures was conducted via Shapiro–Wilk’s normality test. Considering that a large number of variables did not present normal results (see Results section), we decided to proceed with nonparametric methods for the subsequent analyses. Given that the final sample (n = 87) came from two different years (2017 and 2018), the homogeneity of the samples was assessed using the Mann–Whitney U-test alongside cognitive and sociodemographic measures. Chi-square analyses were conducted when the sociodemographic variables were not continuous.

Mean, median and deviation values were calculated for each variable, group, and assessment phase. Likewise, Spearman correlation analyses were conducted within each cognitive task. To determine the impact of the cognitive intervention, statistical analyses were performed in the following order for each assessment task. First, we compared cognitive outcomes between and within experimental groups (INT and CON groups). Between-group comparisons were performed separately for the pre- and post-training assessments using the Mann–Whitney U-test for independent samples. Within-group analyses were performed separately for the CON and INT groups using the Wilcoxon signed-rank test for paired samples.

After comparing the experimental groups, we were interested in comparing the four performance and experimental groups (CON HPG, INT HPG, CON LPG, and INT LPG). We performed Kruskal–Wallis statistical analyses separately for the pre- and post-training assessments to look for differences between the four groups. For significant comparisons, we made planned comparisons using the Mann–Whitney U-test to identify where the differences resided. Planned comparisons were described as the CON vs. INT groups for the HPG and LPG separately (to look for differences between the experimental groups within each performance group) and the HPG vs. LPG for the CON and INT groups separately (to look for differences between performance groups within experimental groups). Finally, within-group analyses were performed separately for the four groups using the Wilcoxon signed-rank test for paired samples. For all measures, effect sizes were calculated. For the Mann–Whitney U-test and the Wilcoxon signed-rank test, the *r-*value was used. For Kruskall–Wallis, Epsilon-squared (*E^2^_R_*) was calculated [68,69].

All statistical procedures were carried out using RStudio 1.1.463 (R version 3.5.3) and the packages “tidyverse”, “psych”, “knitr”, and “rcompanion”.

## 3. Results

### 3.1. Descriptive Statistics

Descriptive statistics, as well as normality and homogeneity tables for sociodemographic, health, cognitive, and temperamental measures are available in the Supplementary Materials (Tables S1–S3). All variables were homogeneous between the intervention, performance, and sample groups.

### 3.2. Stroop Task

The children showed a high number of *omitted trials*, meaning that they did not answer the trials in the given time. Since the source of such a high number of omissions could not be determined. we decided to include these trials in a separate variable of performance by generating two performance variables, i.e., the number of *omitted trials* and *performance* (correct vs. responded), which were both highly correlated with the proportion of total correct trials (correct vs. administered, Tables S6 and S7 in Supplementary Materials). Reaction times (RT) were calculated for each participant by calculating the median RT of all correct trials; in addition, all trials with RT below 250 ms were excluded, as they were considered impulsive responses [56,57].

From the 82 children in the sample, 23 were excluded from the analyses due to technical problems with the tasks, either in the pre- or post-assessment phases. In summary, the sample for the analyses of Stroop Task consisted of 59 children. Group conformation resulted in the following distribution: INT, n = 24; CON, n = 35; low INT, n = 9; low CON, n = 16; high INT, n = 15; high CON, n = 19. For descriptive and statistical tables, see Supplementary Materials (Tables S4, S5 and S8–S15).

#### 3.2.1. Experimental Groups Comparisons

Mann-Whitney U-Test analyses showed no differences between the INT and CON groups in the pre- and post-training assessments. Conversely, the Wilcoxon signed-rank test showed differences within both groups along the assessment phases. *Omitted trials* diminished for the CON group in the incongruent block (*p* = 0.04; *r* = −0.27). *Performance* increased for the CON group in the congruent block (*p* = 0.03; *r* = 0.25) and for the INT group in the incongruent block (*p* = 0.01; *r* = 0.4). Mean RT diminished only for the CON group in the incongruent block (*p =* 0.008; *r* = 0.31). 

#### 3.2.2. Performance Group Comparisons

In the pre-training phase, Kruskal–Wallis analyses showed differences in the number of *omitted trials* in the incongruent block (*p* = 0.007; *E^2^_R_* = 0.20), *performance* in the congruent (*p* < 0.001; *E^2^_R_* = 0.28), incongruent (*p* < 0.001; *E^2^_R_* = 0.31), and mixed blocks (*p* < 0.001; *E^2^_R_* = 0.27), and *RT* in the congruent block (*p* = 0.009; *E^2^_R_* = 0.19). In the post-training phase, analyses showed differences in performance in the mixed block (*p* = 0.01; *E^2^_R_* = 0.17) and particularly in the congruent trials of the mixed block (*p* < 0.02; *E^2^_R_* = 0.16).

According to the planned comparisons in pre-training phase, the number of *omitted trials* was higher in the LPG INT than in the HPG INT group for the incongruent block (*p* = 0.01; *r = 0*.59). *Performance* was lower in the LPG CON than in the LPG INT (*p* = 0.03; *r* = −0.53) and the HPG CON (*p* = 0.003; *r* = 0.56) for the congruent block, lower in the LPG INT than in the HPG INT for the incongruent (*p* = 0.006; *r* = 0.65) and mixed blocks (*p* = 0.02; *r* = 0.56), and lower in the LPG CON than in the HPG CON for the incongruent (*p* = 0.02; *r* = 0.45) and mixed blocks (*p* = 0.01; *r* = 0.5). RT was higher in the LPG INT than in the HPG INT for the congruent block (*p* = 0.02; *r* = 0.57). 

No differences between groups were detected with the planned comparisons in the post-training assessment for any variable, although descriptive values showed that a portion of the LPG in the INT group reached higher performance than those in the CON group (third quartile). Additionally, all groups seemed to decrease their RT in the post-training assessment, with the exception of the LPG INT.

According to the Wilcoxon signed-rank test for paired samples, significant differences were found within both groups along assessment phases. The number of *omitted trials* diminished for the LPG CON in the incongruent block (*p* = 0.03; *r* = 0.40). *Performance* increased for the LPG CON in the congruent block (*p* = 0.01; *r* = 0.53) and for the LPG INT in the incongruent block (*p* = 0.02; *r* = 0.62). RT within the HPG CON diminished in the congruent (*p* = 0.04; *r =* −0.37) and incongruent blocks (*p* = 0.02; *r =* −0.32). The *Spatial incompatibility effect* increased in the mixed block for both the HPG and LPG INT, even though only the latter showed a statistical difference (*p* = 0.03; *r* = 0.46).

### 3.3. Child ANT Task

As in the Stroop Task, participants presented a high number of *omitted trials.* Consequently, the same performance variables were generated, i.e., *omitted trials* and *performance* (correct vs. responded), which were both highly correlated with the proportion of correct total trials (correct vs. administered; Tables S18 and S19 in Supplementary Materials). Reaction times (RT) were calculated for each participant by calculating the median RT of all correct trials; in addition, all trials with RT below 250 ms were excluded as they were considered impulsive responses [56,57].

From the 82 children in the sample, 15 were excluded from the analyses due to technical problems with the task, either in the pre- or post-assessment phases. In summary, the sample for the analyses of the Child ANT consisted of 67 children. Group conformation resulted in the following distribution: INT, n = 30; CON, n = 37; low INT, n = 13; low CON, n = 18; high INT, n = 17; high CON, n = 19. For descriptive and statistical tables, refer to the Supplementary Materials (Tables S16, S17 and S20–S27).

#### 3.3.1. Experimental Group Comparisons

Mann–Whitney U-test showed no significant differences between the INT and CON groups in the pre-training assessments, but did exhibit differences in the post-training assessments. The CON group demonstrated a smaller number of *omitted trials* than the INT group in the overall task (*p* = 0.04; *r* = 0.24) and in the congruent trials (*p* = 0.01; *r* = 0.28). In addition, the INT group showed higher *performance* than the CON in the overall task (*p* = 0.04; *r* = 0.24) and in incongruent trials (*p* < 0.03; *r* = 0.26).

Conversely, the Wilcoxon signed-rank test demonstrated significant differences within both groups along assessment phases. The number of *omitted trials* diminished for CON in the overall task (*p* < 0.001; *r* = 0.43) and in the congruent (*p* = 0.005; *r* = −0.35) and incongruent trials (*p* < 0.001; *r* = −0.42), and diminished for INT only in the incongruent blocks (*p* = 0.03; *r* = −0.33). *Performance* increased for CON in the overall task (*p* = 0.004; *r* = 0.36) and in the congruent (*p* = 0.005; *r* = 0.35) and incongruent trials (*p* = 0.003; *r* = 0.30), and increased for the INT in the total task (*p* < 0.001; *r* = 0.66) and in congruent (*p* < 0.001; *r* = 0.45) and incongruent trials (*p* < 0.001; *r* = 0.65). RT diminished for CON in the congruent trials (*p* = 0.005; *r* = 0.33). The efficiency of the executive network increased for CON (*p* = 0.002; *r* = 0.35) and INT (*p* < 0.001; *r* = 0.51).

#### 3.3.2. Performance Group Comparisons

In the pre-training phase, Kruskal–Wallis analyses showed significant differences in the number of *omitted trials* in the overall task (*p* = 0.009; *E^2^_R_* = 0.17) and in the congruent (*p* = 0.01; *E^2^_R_* = 0.17) and incongruent trials (*p* = 0.01; *E^2^_R_* = 0.15), for RT in the congruent trials (*p* = 0.02; *E^2^_R_* = 0.13), and for the orienting network (*p* = 0.005; *E^2^_R_* = 0.18). In the post-training phase, analyses showed differences in the number of omitted trials in the overall task (*p* = 0.004; *E^2^_R_* = 0.20) and in the congruent (*p* < 0.005; *E^2^_R_* = 0.19) and incongruent trials (*p* = 0.01; *E^2^_R_* = 0.16). *Performance* was significantly different in the total task (*p* = 0.04; *E^2^_R_* = 0.11) and in the incongruent trials (*p* < 0.03; *E^2^_R_* = 0.12). Finally, RT showed differences in the incongruent trials (*p* = 0.04; *r* = 0.12).

According to the planned comparisons in the pre-training phase, the number of *omitted trials* was higher in the LPG INT than in the HPG INT group for the whole task (*p* = 0.008; *r* = 0.53), for the congruent trials (*p* = 0.008; *r* = 0.56), and for incongruent trials (*p* = 0.03; *r* = 0.48). *Performance* was lower in the LPG CON than in the HPG CON for congruent trials (*p* < 0.03; *r* = 0.43). Efficacy of the orienting network was lower in the LPG CON than in the LPG INT (*p* = 0.01; *r* = 0.52) and higher in the LPG CON than in the HPG CON (*p* = 0.005; *r* = 0.53). Efficacy in the executive network was lower in the LPG INT than in the HPG INT (*p* = 0.03; *r* = 0.48). No differences between groups were found according to the planned comparisons in the post-training assessment for any variable. Nonetheless, descriptive values of the total of the trials showed that the LPG CON may have demonstrated lower performance than the other three groups. On the other hand, for the incongruent trials, descriptive values showed that both the HPG and LPG INT tended to perform better than the HPG and LPG CON.

According to the Wilcoxon signed-rank test, differences were found within groups along the assessment phases. The number of *omitted trials* decreased for the LPG CON in the overall task (*p* = 0.02; *r* = 0.47) and also in the incongruent trials (*p* = 0.007; effect Size = −0.49), for the LPG INT in the incongruent trials (*p* = 0.04; *r* = −0.42), and for the HPG CON in the overall task (*p* < 0.005; *r* = 0.47), congruent (*p* = 0.02; *r* = 0.46), and incongruent trials (*p* = 0.002; *r* = 0.41). The number of *omitted trials* decreased for the HPG INT group, but did not show statistical significance. *Performance* increased for the LPG CON in the congruent trials (*p* = 0.04; *r* = 0.38) and for the LPG INT in the overall task (*p* = 0.003; *r* = 0.65) and in congruent (*p* = 0.008; *r* = 0.56) and incongruent trials (*p* = 0.006; *r* = 0.61), for the HPG CON in the overall task (*p* = 0.02; *r* = 0.36) and in congruent (*p* = 0.004; *r* = 0.34) and incongruent trials (*p* = 0.003; *r* = 0.33), and for the HPG INT in the overall task (*p* = 0.001; *r* = 0.67) and in the incongruent trials (*p* < 0.001; *r* = 0.71). RT diminished for the LPG INT in the congruent trials (*p* = 0.007; *r* = 0.46) and for the HPG CON in the whole task (*p* = 0.04; *r* = 0.28) and particularly in the congruent trials (*p* = 0.009; *r* = 0.42). Descriptive statistics also showed a decrease in RT in the incongruent trials for both low-performing groups, especially for LPG INT. The efficacy in executive network increased for all four performance groups, showing statistical differences for the LPG CON (*p* = 0.007; *r* = 0.43), LPG INT (*p* = 0.001; *r* = 0.67), and HPG CON (*p* = 0.03; *r* = 0.27). The *alert network* score diminished for the HPG CON (*p* < 0.05; *r* = 0.33). 

### 3.4. Summary of the Most Important Effects

**Figure 3:**
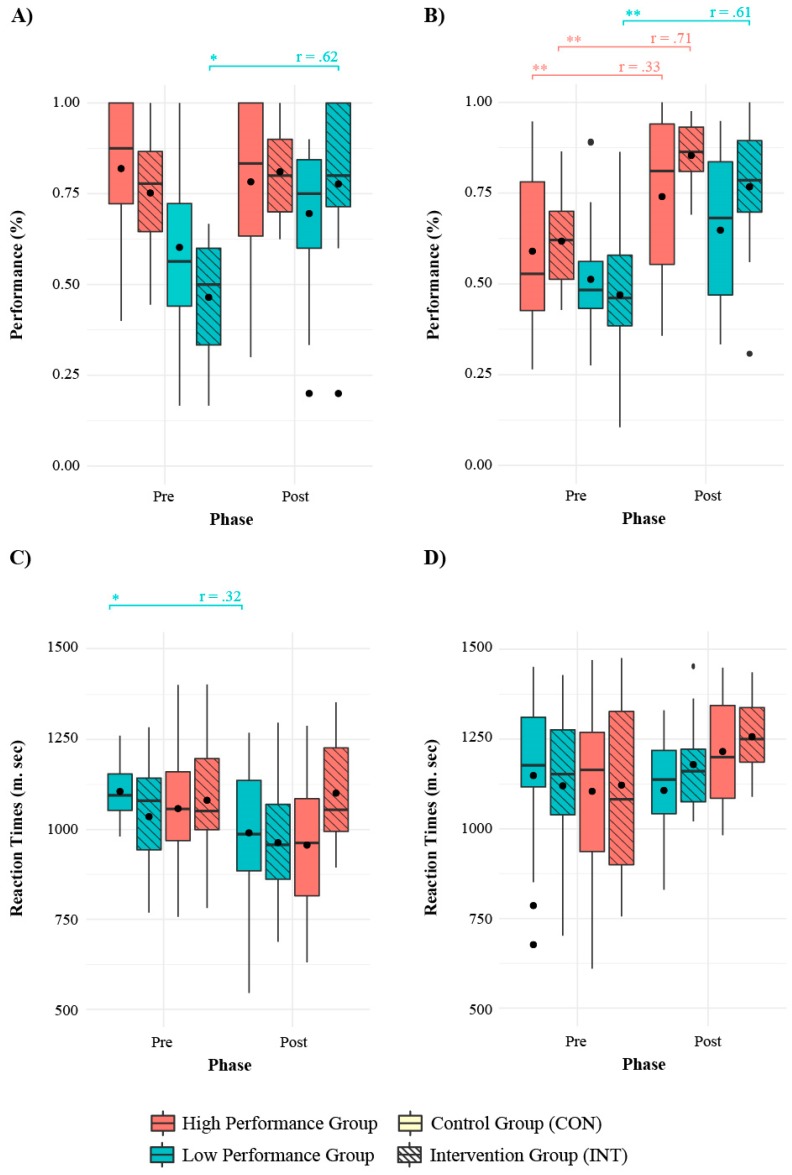
Performance values in pre- and post-training assessments for the performance and experimental groups. (**A**) *Performance* for the incongruent block of the Stroop task; (**B**) RT for the incongruent block of the Stroop task; (**C**) *Performance* for incongruent trials of the Child Attentional Networks Test (ANT); (**D**) RT for incongruent trials of the Child ANT.

The most important effects for both tasks are displayed in Figure 3. In the incongruent block of the Stroop task (Figure 3A,C) the LPG INT benefited the most from training while maintaining the same RT as in the pre-training assessment. Moreover, while the HPG CON decreased RT within the phases, no changes were seen in performance. In the incongruent trials of the Child ANT task (Figure 3B,D), all groups except LPG CON showed within-group increases in performance. Nonetheless, the HPG INT and LPG INT showed the largest differences. In contrast with the Stroop task RT values, no within-group differences were found for the Child ANT task. 

## 4. Discussion

To our knowledge, no other previous study generated different performance profiles from a sample of children and implemented an adapted and adaptive cognitive training intervention for each group. Previous literature focused mostly on the association between individual differences in baseline cognitive measures and training impact, with some studies with children showing how baseline cognitive performance predicted training gains [32,41,46,47]. Nonetheless, in those studies, the same intervention was administered to children without taking these differences into account.

The present study aimed to analyze the impact of a computerized cognitive training intervention adapted to different cognitive profiles in preschoolers from UBN homes. Different interventions were administered to children according to their initial performance in a variety of cognitive tasks. Four groups were constructed according to performance, i.e., high- and low-performers for intervention and control conditions. We hypothesized that children in the intervention groups would perform better than children in the control groups after training. In addition, we aimed to explore impact differences in each performing group. The results showed that the impact of the intervention followed distinct patterns for each performance and experimental group according to the cognitive task.

Overall, the results showed that when analyzing children only by the experimental condition (i.e., before evaluation of the performing groups), the INT group benefited from the intervention; even when the CON group showed improvement (e.g., in the ANT task), the INT group showed a greater effect. On the other hand, while children in the LPG INT clearly benefited the most from the Stroop task intervention, both the HPG and LPG INT groups outranged the HPG and LPG CON groups in the Child ANT task. This effect was higher in the incongruent trials of the task. 

In addition, the RT decreased for the CON group and showed no changes for the INT group in the Stroop task. Also, all groups showed reduced RT, except for the LPG INT group. In the Child ANT task, children in the CON group decreased their initial RT in the congruent trials but not in the incongruent ones. In particular, major decreases in RT were shown for the LPG INT and the HPG CON. In the incongruent trials, although not statistically significant, the LPG INT showed an increase in RT, in line with the number of omitted trial results. In the Stroop, task the CON group, particularly the LPG CON group, showed a decrease in the number of omitted trials. Similarly, in the Child ANT task, the CON group decreased the number of omitted trials in both congruent and incongruent trials, with greater effects shown for the HPG and LPG CON groups. 

Given that one of the instructions in the INT group was to think before acting (e.g., pressing a button), increasing or maintaining RT values may have been an effect of the intervention. On the other hand, considering the high correlation between RT and the number of omitted trials, the CON group might have experienced lower numbers of omitted trials because they responded faster.

In the Stroop task, the spatial incompatibility effect was assessed in the mixed block. In this task, differences within groups were seen within both the high- and low-performing INT groups. In the Child ANT, this measure is known as the executive network; differences were shown within both the CON and INT groups and all four performance groups. Statistical differences were found for all of the groups except the HPG INT group. In both tasks, post-training assessment showed a lower variance within each group toward a positive value, indicating that by the end of the study, the majority of children took longer to respond to the incongruent than the congruent trials. This was the expected behavior in this age given that the incongruent trials impose a higher inhibitory effort than congruent trials [57]. This effect was more pronounced for both of the INT groups in the Stroop take. It should be noted that, in both tasks, the effect sizes were moderate and most differences were found in the within-group analyses, with few of them in the post-training between-group analyses. 

Some limitations of the study included that the final sample resulted in a lower number of children due to difficulties linked to unforeseen events in the families and the institution (e.g., house moving, illness, scholar acts, and daily activity schedules). In addition, the control and intervention groups were unbalanced. The sample size and composition prevented us from generalizing the conclusions. In this sense, more studies addressing individual differences based on pre-training performances to assign control and intervention conditions are necessary. On the other hand, the performance groups for the inhibitory control schemes were conformed based only on the Stroop task. For future studies, a unique compound measure could be made for tasks to assess related domains [1]. Linked to this, the performance groups were distributed according to a median split, which was done due to the need for an easy and fast way of classifying performance. Nonetheless, future studies should explore other techniques, such as clustering or growth models in order to achieve more precise categorization exceeding binary groups such as “high” and “low” [34] and avoid normative terms. The differences between the performance groups´ outcomes may be explained by the differences in the training menus given to each group or their inherent baseline differences. On the other hand, the lack of differences between the groups could be related to the fact that the performing groups were based only on Stroop task performance and performance in the Child ANT task could not be easily inferred from this. Future studies could address these questions with a suitable design.

Given the sociodemographic, cultural, and geographical characteristics of our sample, it would be inconvenient to compare performance levels of other samples presented in the literature. It could be mentioned, however, that effects of the intervention followed a developmental trajectory in accordance with the literature [2,70]. For instance, congruent-alone trials were the easiest, followed by the achievement of good performance in incongruent-alone and lastly correct performance in the mixed condition.

As mentioned previously, different interventions were proven to be effective in changing performance levels in executive demand-based cognitive tasks in children from poor homes over the last few decades [23,25,26,31,32,71]. However, only a few analyzed the effects of individual lab-based training, in which inhibitory control and attentional processes were trained [31,32]. For instance, Goldin and colleagues (2014) found differences in the INT group in both the ANT and Stroop tasks after an adaptive training intervention. Contrary to our study, this effect was significant only for RT; no effects were verified in performance measures. The training activities of that study were the same as in our study, with one difference being that we added adapted schemes for different performance groups to the adaptive training. The children in our study started from and passed through different difficulty levels according to their baseline performances. In addition, children in the intervention implemented by Goldin and colleagues were exposed to a schedule of three sessions per week for a maximum of 27 sessions with a median of 23.94 sessions. In our study, sessions were weekly and we decided to only analyze children who finished 12 sessions. Another source of variation was the age of children in both samples. Thus, differences between these studies could be due to developmental differences or intervention doses.

## 5. Conclusions

This study provides evidence regarding the possible effects of a computerized and lab-based cognitive training intervention in a school context, based on the consideration of individual differences in the design of the training activities. In other words, it contributes toward an experience of a plausible training intervention in which different groups of children were identified based on their cognitive characteristics, assigning different intervention approaches that were designed and implemented for this purpose. This approach could be applied to the identification of populations at risk or with particular needs.

## Supplementary Materials

The following are available online at https://www.mdpi.com/1660-4601/17/8/2912/s1, Table S1. Homogeneity for sociodemographic factors between samples and experimental groups; Table S2. Normality and homogeneity of samples for the STROOP task (Hearts and Flowers); Table S3. Normality and homogeneity of samples for Child ANT; Table S4: STROOP task performance summary data for experimental groups in assessment phases (congruent and incongruent blocks); Table S5: STROOP task performance summary data for experimental groups in assessment phases (mixed block); Table S6: Correlation between variables for the STROOP task (Hearts and Flowers) in congruent and incongruent blocks; Table S7: Correlation between variables for the STROOP task (Hearts and Flowers) in the mixed block; Table S8: STROOP task nonparametric statistical comparison between and within experimental groups; Table S9: STROOP task performance summary data for low-performance experimental groups in assessment phases (congruent and incongruent blocks); Table S10: STROOP task performance summary data for low-performance experimental groups in assessment phases (mixed block); Table S11: STROOP task performance summary data for high-performance experimental groups in assessment phases; Table S12: STROOP task performance summary data for high-performance experimental groups in assessment phases; Table S13: STROOP task nonparametric Wilcoxon statistical comparison within experimental groups for high- and low-performance groups; Table S14: STROOP task Kruskal–Wallis nonparametric statistical comparison between performance and experimental groups for pre- and post-training phases; Table S15: STROOP task nonparametric planned comparisons in pre- and post- training assessments; Table S16: Child ANT performance summary data for experimental groups in assessment phases (general variables); Table S17: Child ANT performance summary data for experimental groups in assessment phases (congruent and incongruent trials); Table S18: Correlation between variables for Child ANT in general variables; Table S19: Correlation between variables for Child ANT in congruent and incongruent trials; Table S20: Child ANT task nonparametric statistical comparison between and within experimental groups; Table S21: Child ANT task performance summary data for low-performance experimental groups in assessment phases; Table S22: Child ANT task performance summary data for low-performance experimental groups in assessment phases; Table S23: Child ANT task performance summary data for high-performance experimental groups in assessment phases; Table S24: Child ANT task performance summary data for high-performance experimental groups in assessment phases; Table S25: Child ANT task Kruskal–Wallis nonparametric statistical comparison between performance and experimental groups for pre- and post-training phases; Table S26: ANT task nonparametric planned comparisons in pre- and post- training assessments; Table S27: Child ANT task nonparametric statistical comparison within experimental groups for high- and low-performance groups.
