# Supplementary material for: Impact of an Individualized Cognitive Training Intervention in Preschoolers from Poor Homes
Source: Int J Environ Res Public Health. 2020 Apr 23;17(8):2912. doi: 10.3390/ijerph17082912 (PMC7215356; doi:10.3390/ijerph17082912)
Supplement: Supplementary file 1 [file ijerph-17-02912-s001.pdf]

Impact of an Individualized Cognitive Training Intervention in Preschoolers from Poor Homes

Supplementary Material

This supplementary material provides descriptive, correlation and comparative tables for both STROOP and Child-ANT task to support reading of the main manuscript.

STROOP Task

Experimental groups comparisons

Table S1. STROOP Task Performance Summary Data for Experimental Groups in assessment phases. (congruent and incongruent blocks).

	n	Pre					Post				
		Mean	SD	Median	Q25	Q75	Mean	SD	Median	Q25	Q75
						**Congruent Trials**					
Omitted Trials											
Intervention	24	0.87	1.07	1.0	0	1	0.87	0.89	1	0	1.25
Control	35	0.91	1.01	1.0	0	1	0.8	1.05	0	0	1.00
Performance											
Intervention	24	0.91	0.12	1	0.85	1	0.9	0.13	1	0.85	1
Control	35	0.86	0.14	0.87	0.75	1	0.91	0.14	1	0.86	1
RT											
Intervention	24	868.28	160.39	824.21	795.06	974.90	901.52	173.16	933.01	741.87	1030
Control	35	879.09	142.22	882	830.95	936.08	840.51	118.15	813.25	770.5	907
						**Incongruent Trials**					
Omitted Trials											
Intervention	24	2.29	1.89	2	0	1	1.79	1.97	1	0	3.25
Control	35	2.11	1.62	2	1	3	1.28	1.58	1	0	2
Performance											
Intervention	24	0.64	0.21	0.66	0.5	0.8	0.79	0.18	0.8	0.7	0.9
Control	35	0.72	0.23	0.75	0.52	0.9	0.74	0.21	0.8	0.6	0.9
RT											
Intervention	24	1052.72	168.11	1065.25	949.45	1168.17	1015.03	183.76	992.57	896.63	1145.82
Control	35	1083.82	127.86	1083.5	996.5	1161.1	975.02	179.8	971.25	823.56	1115.88

Table S2. STROOP Task Performance Summary Data for Experimental Groups in assessment phases. (Mixed Block).

	n	Pre					Post				
		Mean	SD	Median	Q25	Q75	Mean	SD	Median	Q25	Q75
						Mixed Trials					
Omitted Trials											
Intervention	24	6.45	3.36	6.5	5	9	8.04	4.79	7.5	5	11.25
Control	35	6.74	4.19	7.0	4	9	6.14	4.41	6	3	9
Performance											
Intervention	24	0.56	0.19	0.51	0.42	0.66	0.65	0.16	0.63	0.56	0.71
Control	35	0.57	0.18	0.54	0.47	0.66	0.59	0.18	0.57	0.47	0.69
RT											
Intervention	24	1061.53	158.56	1082.91	936.08	1194.28	1104.73	187.66	1114.55	1021.2	1212.02
Control	35	1061.56	179.96	1102.4	995.26	1194.26	1063.92	188.21	1114.87	1012.56	1186.79
Incompatibility Effect											
Intervention	24	−11.25	281.27	0.75	−144	106.43	78.33	158.04	58.66	4.58	130.5
Control	35	63.5	194.66	74.83	−105	183.42	22.28	255.94	−13.82	−161.97	140.08
Congruent trials											
Omitted Trials											
Intervention	24	2.82	1.52	3.0	2.00	4.00	2.90	2.02	2.0	1.00	5.00
Control	35	2.93	2.16	3.0	1.00	4.00	2.00	1.71	2.0	1.00	3.00
Performance											
Intervention	24	0.78	0.23	0.83	0.69	1.00	0.76	0.18	0.75	0.66	0.88
Control	35	0.68	0.27	0.75	0.50	0.88	0.66	0.22	0.66	0.50	0.80
RT											
Intervention	24	1051.73	191.81	1061.00	929.47	1208.33	1051.59	208.53	1042.75	924.60	1174.25
Control	35	1060.90	166.50	1063.22	1009.40	1156.00	1066.45	191.66	1114.50	960.00	1216.16
Incongruent trials											
Omitted Trials											
Intervention	24	3.54	2.24	3.5	2.25	4.75	4.70	2.98	4.0	2.00	7.00
Control	35	3.74	2.44	4.0	1.50	5.00	3.53	2.66	3.0	2.00	5.00
Performance											
Intervention	24	0.46	0.29	0.44	0.23	0.62	0.62	0.26	0.61	0.42	0.86
Control	35	0.50	0.27	0.50	0.30	0.68	0.59	0.28	0.62	0.37	0.83
RT											
Intervention	24	1047.69	292.22	1128.32	844.99	1245.68	1145.13	194.78	1158.36	1082.59	1233.54
Control	35	1106.29	232.36	1150.25	986.50	1263.00	1078.81	252.98	1114.83	958.96	1272.97

Table S3. Correlation between variables for STROOP Task (Hearts and Flowers) in congruent and incongruent blocks.

	1	2	3	4	5
1. Omitted trials	-	−0.78 ***	−0.28	0.11	-
2. Proportion of correct trials (Correct vs. administered)	−0.68 ***	-	0.81 ***	0.17	-
3. Performance (Correct vs. responded)	−0.23	0.85 ***	-	0.39 **	-
4. RT	0.37 *	−0.02	0.2	-	-

Note. RT = Reaction Time; Congruent trials' correlations above diagonal and Incongruent trials' correlations below. *** $p < 0.001$. ** $p < 0.01$. *$p < 0.05$.

Table S4. Correlation between variables for STROOP Task (Hearts and Flowers) in the mixed block.

	1	2	3	4	5
1. Omitted trials	-	0.57 ***	0.07	0.4 *	-0.03
2. Proportion of correct trials (Correct vs. administered)	-	-	0.84 ***	0.14	0.12
3. Performance (Correct vs. responded)	-	-	-	0.4 *	0.14
4. RT	-	-	-	-	0.25
5. Spatial incompatibility effect (Incongruent vs. congruent RT)	-	-	-	-	-

Note RT = Reaction Time. *** p < 0.001. ** p < 0.01. *p < 0.05.

Table S5. Task Non-Parametric Statistical Comparison between and within experimental groups.

	Between						Within					
	Pre			Post			Control			Intervention		
	U	p	r	U	p	r	U	p	r	U	p	r
**Congruent Block**												
Omitted Trials	403	0.78	0.03	457	0.54	-0.08	137.5	0.72	-0.08	70.5	0.91	0.03
Performance	516.5	0.11	-0.2	388.5	0.59	0.07	**77.5**	**0.03**	**0.25**	46	0.6	-0.01
RT	367	0.41	0.1	501	0.21	-0.16	398	0.17	-0.2	125	0.48	0.09
**Incongruent Block**												
Omitted Score	435	0.81	-0.03	471	0.40	-0.1	**328**	**0.04**	**-0.27**	114.5	0.43	-0.15
Performance	337	0.2	0.16	480	0.35	-0.12	241	0.48	0.05	**52**	**0.01**	**0.4**
RT	372	0.46	0.09	466	0.48	-0.09	**476**	**0.008**	**-0.31**	177.5	0.44	-0.12
**Mixed Block**												
Omitted Score	406	0.83	0.02	471	0.11	-0.2	230.5	0.16	-0.2	64.5	0.58	0.08
Performance	387	0.62	0.06	480	0.18	-0.17	168	0.43	0.09	60	0.27	0.17
RT	410	0.88	0.02	466	0.71	-0.04	214	0.81	-0.06	87	0.76	0
Incompatibility Effect	284	0.27	0.14	471	0.23	-0.15	204	0.99	-0.07	66	0.25	0.21
Congruent trials												
Omitted Score	378.50	0.99	0.00	439.00	0.09	0.21	347.50	0.11	0.21	93.50	0.96	0.02
Performance	463.00	0.16	0.18	432.00	0.13	0.19	248.00	1.00	0.07	110.50	0.28	0.22
RT	378.00	0.98	0.00	316.00	0.59	-0.07	259.00	0.70	-0.05	119.00	0.91	0.04
Incongruent trials												
Omitted Score	364.50	0.74	-0.04	473.50	0.13	0.19	606.50	0.56	0.07	213.00	0.26	-0.16
Performance	338.00	0.44	-0.10	408.00	0.69	0.05	460.50	0.21	-0.15	184.00	0.08	-0.26
RT	347.00	0.53	-0.08	436.50	0.38	0.11	582.00	0.78	0.03	229.00	0.44	-0.11

Performance groups comparisons

Table S6. STROOP Task Performance Summary Data for Low-Performance Experimental Groups in assessment phases (Congruent and Incongruent blocks).

	n	Low Performance Group									
		Pre					Post				
		Mean	SD	Median	Q25	Q75	Mean	SD	Median	Q25	Q75
**Congruent Trials**											
Omitted Trials											
Intervention	9	1	1	1	0	1	0.77	0.83	1	0	1
Control	16	1.31	1.19	1	0	2	1.18	1.16	1	0	2
Performance											
Intervention	9	0.91	0.09	0.87	0.85	1	0.97	0.05	1	1	1
Control	19	0.78	0.12	0.81	0.75	0.83	0.89	0.16	0.93	0.85	1
RT											

Intervention	9	988.84	154.71	983.12	877.4	1074.14	969.28	179.01	1013.85	929.16	1098
Control	19	865.69	190.96	870.40	795.76	921.95	873.15	103.44	854.79	792.65	938.01
					Incongruent Trials						
Omitted Trials											
Intervention	9	3.77	1.78	4	2	5	2.11	2.14	1	0	4
Control	19	2.62	1.66	3	1.75	3.25	1.31	1.77	0.5	0	2
Performance											
Intervention	9	0.46	0.17	0.50	0.33	0.6	0.77	0.25	0.8	0.71	1
Control	19	0.6	0.23	0.56	0.44	0.72	0.69	0.2	0.75	0.6	0.84
RT											
Intervention	9	1081.19	187.53	1051.0	999.16	1197	1101.09	172.66	1054.8	994.4	1226.5
Control	19	1058.01	164.45	1056.75	968.83	1160.05	956.45	169.54	962.34	815.61	1085.35

Table S7. STROOP Task Performance Summary Data for Low-Performance Experimental Groups in assessment phases (Mixed block).

	n	Low Performance Group									
		Pre					Post				
		Mean	SD	Median	Q25	Q75	Mean	SD	Median	Q25	Q75
					Mixed Trials						
Omitted Trials											
Intervention	9	7.77	3.76	9	5	11	8.33	6.08	7	5	11
Control	19	8.06	4.56	7.5	5	12.25	7.75	4.9	7.5	3.75	10.25
Performance											
Intervention	9	0.42	0.1	0.41	0.36	0.45	0.56	0.15	0.58	0.5	0.62
Control	19	0.47	0.1	0.5	0.36	0.58	0.51	0.14	0.54	0.4	0.62
RT											
Intervention	9	1019.65	175.4	1039.25	842.42	1152.28	1140.79	234.55	1134.66	1072.93	1215.10
Control	19	996.88	207.62	1059.61	861.09	1100.81	1026.45	203.7	1052.89	991.87	1170.65
Incompatibility Effect											
Intervention	9	-137.28	308.41	-144	-407.43	38.67	106.6	163.6	53.08	32.58	131.74
Control	19	5.4	196.62	6.81	-132.96	83.75	88.62	276.43	47.82	-100.72	280.41
Congruent trials											
Omitted Trials											
Intervention	9	3.00	1.69	3.0	1.75	4.25	3.12	2.35	2.5	1.75	5.00
Control	19	3.71	2.52	3.5	2.00	6.00	2.66	1.83	2.0	1.00	4.00
Performance											
Intervention	9	0.77	0.18	0.80	0.65	0.87	0.69	0.17	0.70	0.62	0.81
Control	19	0.58	0.25	0.61	0.34	0.74	0.57	0.18	0.55	0.47	0.66
RT											
Intervention	9	1042.82	155.33	1024.12	941.77	1138.00	1071.79	234.44	1120.20	999.25	1181.18
Control	19	1042.09	166.02	1038.00	1004.84	1156.00	1003.90	212.52	1013.33	905.12	1168.70
Incongruent trials											
Omitted Trials											
Intervention	9	4.62	2.72	4.5	3.50	7.00	4.55	3.64	4.0	2.00	6.00
Control	19	4.31	2.35	3.5	3.00	6.00	4.53	3.04	4.0	2.00	7.00
Performance											
Intervention	9	0.28	0.20	0.25	0.15	0.33	0.50	0.29	0.41	0.30	0.60
Control	19	0.41	0.20	0.47	0.21	0.50	0.51	0.24	0.54	0.34	0.71

RT										
Intervention	9	943.21	363.47	851.66	762.00	1235.80	1208.24	233.65	1229.50	1103.00	1403.00
Control	19	1017.49	261.06	1040.43	831.23	1178.43	1070.36	278.71	1083.50	988.41	1255.91

Table S8. STROOP Task Performance Summary Data for High-Performance Experimental Groups in assessment phases.

	n	High Performance Group									
		Pre					Post				
		Mean	SD	Median	Q25	Q75	Mean	SD	Median	Q25	Q75
		Congruent Trials									
Omitted Trials											
Intervention	15	0.8	1.14	0	0	1	0.93	0.96	1	0	1.5
Control	19	0.57	0.69	0	0	1	0.47	0.84	0	0	1
Performance											
Intervention	15	0.91	0.14	1	0.87	1	0.86	0.14	0.87	0.77	1
Control	19	0.92	0.13	1	0.87	1	0.93	0.11	1	0.87	1
RT											
Intervention	15	795.94	116.76	808.25	761.59	838.3	860.86	161.95	828.42	716.43	979.83
Control	19	890.38	86.75	883	839.67	948.26	813.02	125.32	804.6	737.37	849.5
		Incongruent Trials									
Omitted Trials											
Intervention	15	1.4	1.35	1	0	2.5	1.6	1.91	1	0	2.5
Control	19	1.68	1.49	1	0.5	3	1.26	1.44	1	0	2.5
Performance											
Intervention	15	0.75	0.14	0.77	0.64	0.86	0.81	0.12	0.8	0.7	0.9
Control	19	0.81	0.18	0.87	0.72	1	0.78	0.21	0.83	0.63	1
RT											
Intervention	15	1035.64	159.68	1079.5	943.3	1142.78	963.39	175.57	957.28	861.37	1069.83
Control	19	1105.56	85.17	1094.6	1052.65	1154.1	990.65	191.17	986.75	884.75	1136.5

Table S9. STROOP Task Performance Summary Data for High-Performance Experimental Groups in assessment phases.

	n	High Performance Group									
		Pre					Post				
		Mean	SD	Median	Q25	Q75	Mean	SD	Median	Q25	Q75
		Mixed Trials									
Omitted Trials											
Intervention	15	5.66	2.94	6	5	8	7.86	4.06	8	5.5	11
Control	19	5.63	3.60	6	2	9	4.78	3.53	5	1.5	7
Performance											
Intervention	15	0.63	0.19	0.6	0.47	0.73	0.71	0.15	0.66	0.61	0.82
Control	19	0.65	0.18	0.66	0.52	0.76	0.66	0.18	0.63	0.55	0.79
RT											
Intervention	15	1086.65	148.05	1128.81	1003.76	1205.55	1083.09	158.37	1065	1009.19	1191.39
Control	19	1116.03	135.65	1132.76	1096.53	1211.01	1095.48	173.31	1160.66	1032.24	1202.04
Incompatibility Effect											
Intervention	15	51.76	254.91	51.01	-125.3	160.34	60.94	158.6	80.52	4.58	130.5

Control	19	106.32	186.76	108.05	-45.09	237.68	-35.76	229.59	-65.78	-190.84	81.54
Congruent trials											
Omitted Trials											
Intervention	15	2.73	1.48	3.0	2.00	4	2.76	1.87	2.0	1.00	4
Control	19	2.36	1.70	2.0	1.00	4	1.44	1.42	1.5	0.00	2
Performance											
Intervention	15	0.79	0.26	0.85	0.75	1.00	0.80	0.17	0.77	0.66	1.00
Control	19	0.76	0.26	0.85	0.70	0.95	0.73	0.22	0.77	0.64	0.89
RT											
Intervention	15	1056.48	213.73	1068.50	957.18	1208.33	1039.16	199.94	1034.12	876.00	1162.25
Control	19	1074.76	170.01	1099.85	1033.56	1135.70	1118.57	160.07	1166.78	1039.66	1236.79
Incongruent trials											
Omitted Trials											
Intervention	15	2.92	1.73	3.0	2.25	4	4.80	2.65	6.0	2.50	7
Control	19	3.26	2.46	4.0	1.00	5	2.64	1.96	2.0	1.00	4
Performance											
Intervention	15	0.56	0.30	0.48	0.44	0.84	0.70	0.22	0.72	0.53	0.86
Control	19	0.58	0.30	0.60	0.38	0.85	0.67	0.30	0.76	0.40	0.92
RT											
Intervention	15	1107.39	237.17	1136.92	1016.62	1245.68	1107.27	164.44	1118.59	1079.70	1191.95
Control	19	1181.08	179.65	1217.42	1084.97	1291.00	1086.26	236.43	1153.75	968.84	1276.88

Table S10. STROOP Task Non-Parametric Wilcoxon Statistical Comparison within experimental groups for High- and Low-Performance Groups.

	**Low-Performance Group**						**High-Performance Group**					
	**Control**			**Intervention**			**Control**			**Intervention**		
	**U**	**p**	**r**	**U**	**p**	**r**	**U**	**p**	**r**	**U**	**p**	**r**
**Congruent Block**												
Omitted Trials	49	0.83	-0.06	13	0.66	-0.09	25.5	0.75	-0.13	25	0.83	0.11
Performance	**14**	**0.01**	**0.53**	4.5	0.23	0.37	25	0.83	0.02	20	0.05	-0.21
RT	63	0.81	-0.01	25	0.81	-0.01	**146**	**0.04**	**-0.37**	37	0.2	0.16
**Incongruent Block**												
Omitted Score	**97**	**0.03**	**-0.4**	25.5	0.05	-0.4	71	0.54	-0.15	32	0.63	-0.01
Performance	40	0.15	0.26	**2**	**0.02**	**0.62**	84	0.74	-0.05	34	0.25	0.2
RT	100	0.1	-0.29	23	1	0.01	**150**	**0.02**	**-0.32**	80	0.26	-0.22
**Mixed Block**												
Omitted Score	36.5	0.78	-0.04	10	1	0	89	0.1	-0.29	25	0.50	0.14
Performance	19	0.12	0.24	6	0.40	0.32	67	0.97	-0.01	29	0.45	0.11
RT	35	0.78	0.16	10	0.55	0.29	86	0.36	-0.19	46	0.61	-0.2
Incompatibility Effect	23	0.22	0.2	**1**	**0.03**	**0.46**	92	0.22	-0.3	40	0.96	0.03
Congruent trials												
Omitted Score	42.00	0.44	0.15	10.00	1.00	-0.06	92.50	0.06	0.32	45.50	1.00	0.05
Performance	36.00	0.84	0.04	21.50	0.64	0.11	67.00	0.71	0.09	37.00	0.35	0.23

	Low-Performance Group						High-Performance Group					
	Control			Intervention			Control			Intervention		
	U	p	r	U	p	r	U	p	r	U	p	r
RT	43.00	0.88	-0.01	12.00	0.41	-0.11	71.00	0.54	-0.19	55.00	0.52	0.13
Incongruent trials												
Omitted Score	38.00	0.62	-0.03	9.50	0.91	0.03	79.50	0.56	0.08	26.00	0.10	-0.29
Performance	32.00	0.20	-0.22	4.00	0.05	-0.36	50.00	0.36	-0.09	28.00	0.13	-0.18
RT	47.00	0.47	-0.21	4.00	0.05	-0.36	98.00	0.32	0.16	62.00	0.57	0.13

Table S11. STROOP Task Kruskal Wallis Non-Parametric Statistical between performance and experimental groups for Pre- and Pos-Training Phases.

	Pre			Post		
	U	p	E^2_R	U	P	E^2_R
**Congruent Block**						
Omitted Score	4.319	0.228	0.074	5.315	0.150	0.091
Performance	**16.357**	**0.000**	**0.282**	5.684	0.128	0.098
RT	**11.371**	**0.009**	**0.196**	5.611	0.132	0.096
**Incongruent Block**						
Omitted Score	**12.077**	**0.007**	**0.208**	1.180	0.757	0.020
Performance	**18.224**	**0.000**	**0.314**	3.287	0.349	0.056
RT	1.727	0.630	0.029	3.751	0.289	0.064
**Mixed Block**						
Omitted Score	4.494	0.212	0.077	5.869	0.118	0.101
Performance	**16.198**	**0.001**	**0.279**	**10.049**	**0.018**	**0.173**
RT	5.829	0.120	0.101	2.851	0.415	0.049
Incompatibility Effect	5.517	0.137	0.095	3.492	0.321	0.060
Congruent trials						
Omitted Score	2.972	0.395	0.051	6.265	0.099	0.108
Performance	6.888	0.075	0.119	**9.406**	**0.024**	**0.162**
RT	1.097	0.777	0.018	3.370	0.337	0.058
Incongruent trials						
Omitted Score	3.81	0.28	0.06	5.86	0.11	0.10
Performance	7.44	0.05	0.12	5.48	0.13	0.09

	Pre			Post		
	**U**	**p**	E^2_R	**U**	**P**	E^2_R
RT	5.21	0.15	0.08	2.62	0.45	0.04

Table S12. STROOP Task Non-Parametric planned comparisons in pre- and post- training assessments.

	Pre												Post											
	LPG INT vs LPG CON			HPG INT vs HPG CON			HPG INT vs LPG INT			HPG CON vs LPG CON			Low INT vs Low CON			High INT vs High CON			High INT vs LOW INT			High CON vs Low CON		
	U	p	r	U	p	r	U	p	r	U	p	r	U	p	r	U	p	r	U	p	r	U	p	r
**Congruent Block**																								
Omitted Score	61.5	1	-0.12	149	1	0.04	79	1	0.15	207	0.21	0.32	59	1	-0.15	185	0.41	0.28	62.5	1	-0.06	212.5	0.12	0.37
Performance	**118.5**	**0.03**	**0.53**	133	1	-0.06	56	1	-0.15	**54**	**0.003**	**-0.56**	95	0.59	0.29	95.5	0.29	-0.3	97	0.23	0.39	120	0.94	-0.2
RT	100	0.47	0.31	70	0.05	-0.43	**115**	**0.02**	**0.57**	129	1	-0.12	101	0.42	0.32	163	1	0.12	93	0.54	0.3	195	0.63	0.24
**Incongruent Block**																								
Omitted Score	98.5	0.54	0.30	128	1	-0.08	**115.5**	**0.016**	**0.59**	202	0.37	0.28	88	1	0.19	149.5	1	0.04	78	1	0.13	146	1	-0.03
Performance	48	0.72	-0.27	103	0.69	-0.23	**14**	**0.006**	**-0.65**	**70.5**	**0.02**	**-0.45**	93.5	0.93	0.24	139.5	1	-0.01	71	1	0.04	108.5	0.6	-0.24
RT	78	1	0.06	110	1	-0.19	77	1	0.11	122	1	-0.16	104	0.29	0.36	127	1	-0.09	96	0.37	0.34	135	1	-0.09
**Mixed Block**																								
Omitted Score	69	1	-0.03	138	1	-0.02	92	0.56	0.3	197.5	0.53	0.2	76	1	0.04	206	0.11	0.37	64	1	-0.04	206	0.3	0.3
Performance	56.5	1	-0.17	128	1	-0.08	**21.5**	**0.02**	**-0.56**	**61.5**	**0.01**	**-0.5**	85	1	0.14	166	1	0.14	32.5	0.15	-0.42	90	0.16	-0.34
RT	71	1	-0.01	129	1	-0.08	49	1	-0.22	83	0.09	-0.38	96	0.73	0.27	123	1	-0.11	86	1	0.22	111	0.71	-0.22
Incompatibility Effect	32	0.87	-0.25	108	1	-0.15	28	0.5	-0.32	89	0.45	-0.27	61	1	0.06	137	0.61	0.24	56	1	0.05	143	0.81	0.21
Congruent trials																								
Omitted Score	45.00	1	-0.15	160.50	1	0.10	66.00	1	0.08	176.00	0.47	0.26	67.50	1	0.09	165.00	0.20	0.33	56.50	1	0.06	188.00	0.21	0.33
Performance	80.50	0.40	0.33	157.50	1	0.09	46.50	1	-0.18	77.50	0.17	-0.34	83.50	0.54	0.30	130.00	1	0.09	36.00	1	-0.23	73.50	0.10	-0.37
RT	51.00	1	-0.06	144.00	1	0.00	52.00	1	-0.10	113.50	1	-0.12	72.00	1	0.15	84.00	0.77	-0.22	61.00	1	0.13	89.00	0.39	-0.28
Incongruent trials																								
Omitted Score	-	-	-	-	-	-	-	-	-	-	-	-	-	-	-	-	-	-	-	-	-	-	-	-
Performance	-	-	-	-	-	-	-	-	-	-	-	-	-	-	-	-	-	-	-	-	-	-	-	-

	Pre												Post											
	LPG INT vs LPG CON			HPG INT vs HPG CON			HPG INT vs LPG INT			HPG CON vs LPG CON			Low INT vs Low CON			High INT vs High CON			High INT vs LOW INT			High CON vs Low CON		
	U	p	r	U	p	r	U	p	r	U	p	r	U	p	r	U	p	r	U	p	r	U	p	r
RT	-	-	-	-	-	-	-	-	-	-	-	-	-	-	-	-	-	-	-	-	-	-	-	-

Child-ANT Task

Experimental groups comparisons

Table S13. Child-ANT Performance Summary Data for Experimental Groups in assessment phases.

	n	Pre					Post				
		Mean	SD	Median	Q25	Q75	Mean	SD	Median	Q25	Q75
						General Variables					
Omitted Trials											
Intervention	30	25.9	13.09	24.5	15.5	32.75	19.73	12.11	15.5	11	28.5
Control	37	23.72	12.14	20	15	28	13.81	8.61	12	8	19
Performance											
Intervention	30	0.70	0.12	0.69	0.61	0.78	0.88	0.08	0.90	0.83	0.94
Control	37	0.70	0.14	0.66	0.61	0.82	0.81	0.13	0.84	0.70	0.92
RT											
Intervention	30	1123.11	171.22	1119.00	1012.00	1217.31	1134.38	130.01	1114.12	1042.68	1224.12
Control	37	1121.27	180.87	1139.00	1041.75	1245.75	1090.19	138.98	1093.75	992.50	1212.50
Alert Network											
Intervention	30	114.70	147.66	107.25	60.87	222.62	80.78	114.36	103.75	13.00	136.87
Control	37	110.68	175.21	110.00	-14.50	189.00	89.91	151.01	104.00	22.50	164.00
Orienting Network											
Intervention	30	-27.54	129.18	-47.87	-121.93	74.56	33.41	104.54	23.25	-40.18	115.62
Control	37	-9.98	121.90	-0.25	-115.00	78.50	-6.41	123.98	20.00	-56.00	56.50
Executive Network											
Intervention	30	-5.03	174.36	18.5	-82.12	128.12	156.86	82.29	163.0	144.25	197.50
Control	37	13.45	192.90	67.0	-90.50	122.00	138.47	106.36	135.5	104.00	208.00

Table S14. Child-ANT Performance Summary Data for Experimental Groups in assessment phases.

	n	Pre					Post				
		Mean	SD	Median	Q25	Q75	Mean	SD	Median	Q25	Q75
						Congruent Trials					
Omitted Trials											
Intervention	30	9.76	6.21	9.5	5.0	12.00	8.56	6.4	7.0	4.0	11.75
Control	37	9.13	6.69	7.0	5.0	11.00	5.21	4.15	4.0	2.0	8.00
Performance											
Intervention	30	0.82	0.14	0.85	0.72	0.94	0.94	0.05	0.95	0.91	0.99
Control	37	0.82	0.14	0.87	0.76	0.93	0.91	0.10	0.95	0.86	1.00

	n	Pre					Post				
		Mean	SD	Median	Q25	Q75	Mean	SD	Median	Q25	Q75
RT											
Intervention	30	1125.63	166.69	1134	1044.25	1201.12	1055.95	146.03	1038	960.00	1175.37
Control	37	1114.54	161.89	1156	1047.00	1212.00	1020.95	140.68	1011	916.00	1129.50
Incongruent Trials											
Omitted Trials											
Intervention	30	16.13	7.52	16.0	9.5	19.00	11.16	6.76	9.5	6.0	15.25
Control	37	14.59	6.77	13.0	9.0	19.00	8.59	5.06	9.0	5.0	11.00
Performance											
Intervention	30	0.55	0.17	0.53	0.45	0.66	0.81	0.14	0.84	0.75	0.91
Control	37	0.55	0.20	0.48	0.42	0.72	0.69	0.21	0.73	0.48	0.87
RT											
Intervention	30	1120.59	214.58	1126.75	934.37	1302.37	1212.81	125.95	1190.25	1111.50	1326.00
Control	37	1128.00	240.46	1177.00	947.00	1272.00	1159.43	156.52	1159.00	1048.00	1280.00

Table S15. Correlation between variables for Child-ANT in general variables.

	1	2	3	4	5	6	7
1. Omitted trials	-	-	-	-	-	-	-
2. Proportion of correct trials (Correct vs. administered)	-0.73 ***	-	-	-	-	-	-
3. Performance (Correct vs. responded)	-0.1	0.74 ***	-	-	-	-	-
4. RT	0.55 ***	-0.02	0.49 ***	-	-	-	-
5. Orienting Network	0.11	0.07	-0.17	0.28	-	-	-
6. Alerting Network	0	-0.09	-0.12	-0.19	-0.08	-	-
7. Executive Network	-0.11	0.44 **	0.53 ***	0.39 *	0	-0.21	-

Note. *** $p < 0.001$. ** $p < 0.01$. *$p < 0.05$.

Table S16. Correlation between variables for Child-ANT in congruent and incongruent trials.

	1	2	3	4
1. Omitted trials	-	-0.76 ***	-0.15	0.56 ***
2. Proportion of correct trials (Correct vs. administered)	-0.65 ***	-	0.75 ***	-0.11
3. Performance (Correct vs. responded)	-0.19	0.85 ***	-	0.37 **
4. RT	0.43 **	0.05	0.32 *	-

Note: Congruent trials' correlations above diagonal and Incongruent trials' correlations below. *** $p < 0.001$. ** $p < 0.01$. *$p < 0.05$.

Table S17. Child-ANT Task Non-Parametric Statistical Comparison between and within experimental groups.

	Between						Within					
	Pre			Post			Control			Intervention		
	U	p	r	U	p	r	U	p	r	U	p	r
**Congruent Block**												
Omitted Trials	612.5	0.47	0.08	**740**	**0.01**	**0.28**	**444**	**0.003**	**-0.35**	268.5	0.46	-0.12
Performance	548	0.93	-0.01	577	0.78	0.03	**146**	**0.005**	**0.35**	**41**	**<.001**	**0.45**
RT	547	0.92	-0.01	612.5	0.47	0.08	**508**	**0.01**	**-0.33**	311	0.1	-0.24
**Incongruent Block**												
Omitted Trials	615	0.45	0.09	664	0.17	0.16	**590.5**	**<.001**	**-0.42**	**295**	**0.037**	**-0.33**
Performance	594	0.62	0.06	**723.5**	**0.03**	**0.26**	**145**	**0.003**	**0.3**	**27**	**<.001**	**0.65**
RT	537.5	0.83	-0.02	663	0.17	0.16	342.5	0.89	-0.005	154.5	0.11	0.22
**General Variables**												
Omitted Trials	602	0.55	0.07	**714**	**0.04**	**0.24**	**556**	**<.001**	**-0.43**	312	0.1	-0.25
Performance	561	0.94	0	**711.5**	**0.04**	**0.24**	**162**	**0.004**	**0.36**	**13**	**<.001**	**0.66**
RT	528.00	0.73	-0.04	636.00	0.31	0.12	0.31	419.00	0.14	0.56	204.00	-0.02
Alert Network	598.5	0.58	0.06	524	0.7	-0.04	396	0.5	0.006	265	0.51	-0.14
Orienting Network	509.5	0.57	-0.07	635	0.31	0.12	310	0.53	0.03	159	0.13	0.23
Executive Network	503.5	0.52	-0.07	648.5	0.24	0.14	**154**	**0.002**	**0.35**	**55**	**<.001**	**0.51**

Performance groups comparisons

Table S18. Child-ANT Task Performance Summary Data for Low-Performance Experimental Groups in assessment phases.

		Low Performance Group									
	n	Pre					Post				
		Mean	SD	Median	Q25	Q75	Mean	SD	Median	Q25	Q75
General Variables											
Omitted Trials											
Intervention	13	34.30	13.10	32.0	25.00	46.00	22.92	8.46	24.0	14.00	30.00
Control	18	25.72	10.84	26.5	19.25	29.50	16.66	7.31	14.0	11.00	20.75
Performance											
Intervention	13	0.66	0.12	0.63	0.60	0.71	0.86	0.11	0.90	0.81	0.93
Control	18	0.65	0.12	0.63	0.58	0.70	0.76	0.14	0.78	0.67	0.88
RT											
Intervention	13	1173.63	174.39	1123.75	1059.75	1317.00	1170.90	112.28	1199.50	1094.50	1256.50

	n	Low Performance Group									
		Pre					Post				
		Mean	SD	Median	Q25	Q75	Mean	SD	Median	Q25	Q75
Control	18	1105.18	203.61	1163.62	1042.00	1234.87	1136.70	113.47	1125.62	1032.37	1226.56
Alert Network											
Intervention	13	147.76	84.77	136.50	95.00	203.50	57.38	143.58	102.00	-25.50	129.50
Control	18	28.08	138.30	17.75	-76.37	84.87	85.97	198.65	117.75	-11.12	167.37
Orienting Network											
Intervention	13	-52.53	127.01	-49.75	-126.00	13.25	7.67	107.99	2.50	-57.00	42.00
Control	18	-30.52	114.98	-32.62	-129.37	53.62	-46.86	150.44	-29.00	-115.00	56.56
Executive Network											
Intervention	13	-103.96	193.08	-82.50	-222.00	17.00	172.03	92.23	188.50	168.00	206.00
Control	18	-1.13	210.36	54.75	-117.12	118.12	156.08	126.41	162.00	124.12	228.62

Table S19. Child-ANT Task Performance Summary Data for Low-Performance Experimental Groups in assessment phases.

		Low Performance Group									
		Pre					Post				
	n	Mean	SD	Median	Q25	Q75	Mean	SD	Median	Q25	Q75
**Congruent Trials**											
Omitted Trials											
Intervention	13	13.84	6.36	12.0	10.00	18.00	9.15	4.21	10.0	6.00	12.00
Control	18	9.66	5.75	8.0	7.00	13.25	6.55	3.95	6.0	4.00	8.75
Performance											
Intervention	13	0.80	0.15	0.83	0.65	0.95	0.95	0.05	0.97	0.91	1.00
Control	18	0.77	0.14	0.78	0.63	0.91	0.87	0.12	0.90	0.81	0.97
RT											
Intervention	13	1225.61	127.24	1189.50	1132.00	1307.00	1084.88	128.57	1068.00	998.00	1198.50
Control	18	1105.75	191.99	1160.50	1076.37	1211.12	1058.66	111.58	1079.75	961.25	1126.87
**Incongruent Trials**											
Omitted Trials											
Intervention	13	20.46	7.33	19.0	17.00	26.00	13.76	6.01	13.0	9.00	19.00
Control	18	16.05	6.12	17.0	11.25	19.75	10.11	4.02	9.5	6.25	12.00
Performance											
Intervention	13	0.46	0.19	0.46	0.38	0.57	0.76	0.18	0.78	0.69	0.89
Control	18	0.51	0.18	0.48	0.43	0.56	0.64	0.20	0.68	0.46	0.83

		Low Performance Group									
		Pre					Post				
	n	Mean	SD	Median	Q25	Q75	Mean	SD	Median	Q25	Q75
RT											
Intervention	13	1121.65	251.54	1082.50	900.00	1327.00	1256.92	113.74	1250.00	1186.00	1338.00
Control	18	1104.61	261.11	1164.25	936.50	1268.75	1214.75	145.91	1199.50	1085.25	1343.87

Table S20. Child-ANT Task Performance Summary Data for High-Performance Experimental Groups in assessment phases.

		High Performance Group									
	n	Pre					Post				
		Mean	SD	Median	Q25	Q75	Mean	SD	Median	Q25	Q75
General Variables											
Omitted Trials											
Intervention	17	19.47	8.98	17.0	14.00	26.00	17.29	14.06	13.0	8.00	16.00
Control	19	21.84	13.26	19.0	12.00	26.50	11.10	9.05	11.0	3.50	13.00
Performance											
Intervention	17	0.73	0.11	0.71	0.67	0.80	0.89	0.06	0.90	0.84	0.95
Control	19	0.74	0.15	0.73	0.64	0.87	0.85	0.12	0.85	0.75	0.97
RT											
Intervention	17	1084.48	163.30	1114.25	1004.50	1211.50	1106.45	138.80	1110.25	1004.00	1214.00
Control	19	1136.51	160.53	1118.25	1058.00	1273.62	1046.13	149.20	1061.25	934.00	1155.62
Alert Network											
Intervention	17	89.41	180.44	89.50	-15.00	227.50	98.67	86.36	115.50	65.50	138.50
Control	19	188.94	173.33	164.00	119.50	255.25	93.65	91.16	98.00	22.50	151.75
Orienting Network											
Intervention	17	-8.42	131.35	1.75	-75.00	78.25	53.10	100.56	60.50	-29.50	117.50
Control	19	9.47	128.12	26.75	-83.62	121.87	31.89	78.63	29.75	-11.50	56.25
Executive Network											
Intervention	17	70.61	114.48	84.50	10.00	159.50	145.26	74.59	154.50	142.00	163.50
Control	19	27.28	179.49	90.00	-23.00	133.75	121.78	83.24	112.50	101.00	157.0

Table S21. Child-ANT Task Performance Summary Data for High-Performance Experimental Groups in assessment phases.

		High Performance Group									
	n	Pre					Post				
		Mean	SD	Median	Q25	Q75	Mean	SD	Median	Q25	Q75
**Congruent Trials**											
Omitted Trials											
Intervention	17	6.64	3.98	6.0	4.00	10.00	8.11	7.77	5.0	3.00	8.00
Control	19	8.63	7.60	7.0	4.00	10.50	3.94	4.02	2.0	1.00	4.50
Performance											
Intervention	17	0.83	0.15	0.88	0.79	0.93	0.93	0.06	0.95	0.91	0.97
Control	19	0.88	0.12	0.93	0.81	0.97	0.95	0.05	0.96	0.92	1.00
RT											
Intervention	17	1049.17	154.55	1061.50	991.00	1148.00	1033.82	158.28	1034.00	933.00	1092.50
Control	19	1122.86	132.12	1073.00	1019.00	1219.00	985.23	158.28	985.00	877.50	1127.00
**Incongruent Trials**											
Omitted Trials											
Intervention	17	12.82	5.95	11.0	9.00	18.00	9.17	6.78	7.0	4.00	11.00
Control	19	13.21	7.22	9.0	8.50	16.50	7.15	5.62	6.0	3.00	10.50
Performance											
Intervention	17	0.61	0.13	0.62	0.51	0.70	0.85	0.08	0.86	0.80	0.93
Control	19	0.58	0.21	0.52	0.42	0.78	0.74	0.21	0.81	0.55	0.94
RT											
Intervention	17	1119.79	189.74	1152.50	1039.00	1276.00	1179.08	127.52	1160.50	1075.00	1222.00
Control	19	1150.15	224.03	1177.00	1116.50	1311.0	1107.02	151.43	1137.00	1042.00	1218.75

Table S22. Child-ANT Task Kruskal Wallis Non-Parametric Statistical between performance and experimental groups for Pre- and Pos-Training Phases.

	Pre			Post		
	U	p	r	U	p	r
**Congruent Block**						
Omitted Trials	**11.3**	**0.01**	**0.17**	**12.65**	**0.005**	**0.19**
Performance	6.85	0.07	0.1	5.64	0.13	0.08
RT	**9.09**	**0.02**	**0.13**	4.15	0.24	0.06
**Incongruent Block**						
Omitted Trials	**10.286**	**0.016**	**0.156**	**10.61**	**0.01**	**0.16**

	Pre			Post		
	**U**	**p**	**r**	**U**	**p**	**r**
Performance	7.044	0.070	0.107	**8.47**	**0.03**	**0.12**
RT	0.384	0.943	0.005	**8.21**	**0.04**	**0.12**
**General Variables**						
Omitted Trials	**11.48**	**0.009**	**0.17**	**13.2**	**0.004**	**0.20**
Performance	7.29	0.06	0.11	**7.82**	**0.04**	**0.11**
RT	1.75	0.62	0.02	6.47	0.09	0.09
Alert Network	1.94	0.58	0.02	4.91	0.17	0.07
Orienting Network	**12.45**	**0.005**	**0.18**	0.77	0.85	0.01
Executive Network	7.01	0.07	0.1	4.37	0.22	0.06

Table S23. ANT Task Non-Parametric planned comparisons in pre- and post- training assessments.

	Pre												Post											
	LPG INT vs LPG CON			HPG INT vs HPG CON			HPG INT vs LPG INT			HPG CON vs LPG CON			LPG INT vs LPG CON			HPG INT vs HPG CON			HPG INT vs LPG INT			HPG CON vs LPG CON		
	U	p	r	U	p	r	U	p	r	U	p	r	U	p	r	U	p	r	U	p	r	U	p	r
**Congruent Block**																								
**Omitted Trials**	162.5	0.28	0.32	149	1	-0.06	**184**	**0.008**	**0.56**	209	1	0.19	162	0.29	0.32	226.5	0.15	0.34	142	0.77	0.24	250	0.06	0.39
**Performance**	137	1	0.14	130	1	-0.16	98	1	-0.09	**85**	**0.03**	**-0.43**	159.5	0.35	0.3	118	0.66	-0.23	134	1	0.18	105	0.17	-0.33
**RT**	156	0.49	0.28	120.5	0.79	-0.21	**178**	**0.02**	**0.51**	181.5	1	0.05	131	1	0.1	183	1	0.11	136.5	1	0.19	218	0.63	0.23
**Incongruent Block**																								
**Omitted Trials**	155.5	0.50	0.27	164	1	0.01	**173.5**	**0.03**	**0.48**	221	0.52	0.25	158.5	0.39	0.29	196.5	1	0.18	161.5	0.13	0.39	236	0.19	0.32
**Performance**	101.5	1	-0.11	187	1	0.13	52.5	0.06	-0.44	141.5	1	-0.14	158	0.41	0.29	193.5	1	0.16	82	0.96	-0.21	123	0.59	-0.24
**RT**	126	1	0.06	141	1	-0.1	113	1	0.01	157	1	-0.06	137	1	0.14	201	0.86	0.2	153.5	0.30	0.32	232	0.26	0.30
**Mixed Block**																								
**Omitted Trials**	158	0.41	0.29	152	1	-0.05	**181**	**0.01**	**0.53**	220	0.55	0.24	170	0.14	0.38	211	0.47	0.26	160	0.15	0.37	248	0.07	0.38
**Performance**	120.5	1	0.02	149	1	-0.06	71	0.41	-0.3	102	0.14	-0.34	164	0.25	0.33	178.5	1	0.08	104.5	1	-0.04	109	0.24	-0.31
**Alert Network**	109	1	-0.05	148	1	-0.07	92	1	-0.14	138	1	-0.16	136	1	0.13	190	1	0.15	82	0.96	-0.21	122	0.56	-0.24
**Orienting Network**	**190**	**0.01**	**0.52**	113	0.51	-0.25	134	1	0.18	**65**	**0.005**	**-0.53**	100	1	-0.12	171	1	0.05	92	1	-0.14	179	1	0.04
**Executive Network**	81	0.62	-0.25	173.5	1	0.06	**47**	**0.03**	**-0.48**	155.5	1	-0.07	132	1	0.1	204	0.73	0.22	147	0.52	0.27	217	0.66	0.23

Table S24. Child-ANT Task Non-Parametric Statistical Comparison within experimental groups for High- and Low-Performance Groups.

	**Low-Performance Group**						**High-Performance Group**					
	**Control**			**Intervention**			**Control**			**Intervention**		
	**p**	**U**	**r**	**p**	**U**	**r**	**p**	**U**	**r**	**p**	**U**	**r**
**Congruent Block**												
Omitted Trials	0.08	113	-0.29	0.11	68.5	0.37	**0.02**	**138**	**0.46**	1	76.5	0.032
Performance	**0.04**	**39**	**0.38**	**0.008**	**5**	**-0.56**	**0.004**	**10**	**-0.34**	0.05	35	-0.37
RT	0.19	116	-0.31	**0.007**	**84**	**0.46**	**0.009**	**160**	**0.42**	0.81	82	0.09
**Incongruent Block**												
Omitted Trials	**0.007**	**133.5**	**-0.49**	**0.04**	**75**	**0.42**	**0.002**	**156**	**0.41**	0.13	87	0.35
Performance	0.07	44	0.29	**0.006**	**6**	**-0.61**	**0.003**	**22**	**-0.33**	**<.001**	**3**	**-0.71**
RT	0.11	49	0.15	0.12	23	-0.24	0.26	123	0.17	0.6	65	-0.15
**General Variables**												
Omitted Trials	**0.02**	**126**	**-0.47**	0.06	72	0.41	**0.005**	**136**	**0.47**	0.4	94.5	0.24
Performance	0.05	41	0.39	**0.003**	**3**	**-0.65**	**0.02**	**40**	**-0.36**	**0.001**	**10**	**-0.67**
RT	0.82	80.00	0.01	0.88	43.00	-0.02	**0.04**	**145.00**	**0.28**	0.74	69.00	-0.02
Alert Network	0.05	41	0.39	0.12	68	0.31	**0.04**	**146**	**0.33**	0.77	70	-0.01
Orienting Network	0.69	95	0	0.4	33	-0.21	0.43	75	-0.04	0.15	46	-0.22
Executive Network	**0.007**	**24**	**0.43**	**0.001**	**0**	**-0.67**	**0.03**	**42.5**	**-0.27**	0.058	36	-0.34
